# A Case of Life-Threatening Abdominal Wall Hematoma Formation in a Patient on Warfarin Therapy with Concurrent Influenza Infection

**DOI:** 10.7759/cureus.52262

**Published:** 2024-01-14

**Authors:** Bhavani Nagendra Papudesi, Isabella M Alvarado, Parneet Kaur, Srikrishna V Malayala, Sivakoti N Guda, Mathew Mathew, Sai Deepika Potluri

**Affiliations:** 1 Internal Medicine, Suburban Community Hospital, Philadelphia, USA; 2 Medicine, Philadelphia College of Osteopathic Medicine, Philadelphia, USA; 3 Emergency, Civil Hospital, Mukerian, IND; 4 Internal Medicine, Physicians for American Healthcare Access, Philadelphia, USA; 5 Internal Medicine, Medical University of South Carolina, Florence, USA

**Keywords:** covid-19, influenza, hematoma, warfarin, case report

## Abstract

Warfarin therapy provides extensive antithrombotic benefits and, thus, is widely used in the general population. However, as with most medications, there are also risks associated with warfarin use. Specifically, because of the narrow therapeutic window of this drug, patients taking it are at a much higher risk of accidental bleeding. Additionally, patients may also present with bleeding complications when infected with illnesses with coughing as a symptom, such as influenza or COVID-19. These patients have the potential to suffer hemorrhagic morbidities related to the increased intra-abdominal and intra-thoracic pressures that are generated from coughing. Moreover, a synergistic effect is seen when patients find themselves in a situation where they are taking anticoagulation therapy and become infected with illnesses such as influenza or COVID-19. We present a case in which an individual on warfarin therapy was infected with Influenza A. This combination of factors eventually led to massive hemorrhage and large abdominal wall hematoma formation. This case brings to light the importance of having a low threshold for considering the prospect of massive hemorrhage in any patient who is anticoagulated and develops a condition that is associated with increased abdominal pressure. Because these bleeding events can have devastating effects, raising awareness of this risk is increasingly important. Early detection of massive hemorrhage will lead to better outcomes and can ultimately be life-saving for these patients.

## Introduction

While warfarin offers antithrombotic advantages, it may result in severe, life-threatening bleeding in certain patients owing to its narrow therapeutic range. In fact, approximately 10% of those undergoing anticoagulation therapy experience hemorrhagic complications. Most often, both traumatic and spontaneous bleeding occur in the skin, genitourinary, gastrointestinal, spinal, or intracranial regions. In rare cases, this bleeding may even result in hematoma formation if it occurs in specific locations within the body. Most cases include retropharyngeal hematomas, abdominal wall hematomas, esophageal hematomas, and breast hematomas [1]. Cases of retropharyngeal hematomas are often discussed due to their potential to rapidly cause airway obstruction [2]. However, cases of patients on warfarin therapy who develop abdominal wall hematomas specifically are rarely discussed.

The antithrombotic effects of warfarin are not the only possible causes of hematomas. Another cause includes episodes of greatly increased pressure within the body cavity. For example, each time a patient coughs, a transient pressure is created against the glottis. The glottis initially closes following fast and deep inspiration. Then, the thoracic and abdominal muscles contract, which creates an increase in both lung and subglottic pressures. The rapid changes in pressure and energy that occur during a cough lead to unintended and undesirable consequences. In a severe cough, intrathoracic pressures can increase up to 300 mmHg. The expiratory phase is accompanied by hemodynamic changes as well, with the systolic pressure rising to 140 mmHg [3]. Additionally, violent coughing has been reported specifically as an inciting cause of upper airway hematomas, a rare but potentially fatal complication of warfarin anticoagulation [4].

Hematoma formation in the rectus abdominis is a multi-step process. This muscle is formed by two vertically aligned muscles that are separated by the arcuate line into superior and inferior portions. Above the arcuate line, the muscle is enclosed within the aponeuroses of both the external and internal oblique muscles and the transversalis muscle. These aponeuroses are all very strong and provide great protection to the muscle segments. Below the arcuate line, the aponeuroses provide only anterior protection. The posterior sheath separating the rectus muscle from the abdominal compartment consists only of the weak transversalis fascia and the peritoneum. Both the superior and inferior epigastric arteries run between the posterior rectus abdominis muscle and the rectus sheath, where they form anastomoses near the umbilicus. Due to the anatomy of the muscle, aponeuroses, and positioning of the vasculature, hematomas most frequently form in the lower segment, posterior to the muscle. Additionally, the lower segment has the greatest contraction during muscle shortening because it is the longest section. These factors contribute to the higher risk of injury and hematoma formation in this segment, particularly after violent muscle contraction or trauma [5].

Although often a challenge to diagnose, abdominal wall hematomas, such as those within the rectus sheath, may reveal a palpable, non-pulsatile abdominal mass on physical examination. Additionally, they may be accompanied by signs of hemodynamic compromise, including tachycardia and hypotension. More rare signs would include Cullen’s and Grey-Turner’s signs, which are periumbilical and flank ecchymosis, respectively, and would indicate considerable retroperitoneal hemorrhage. Typically, these physical exam findings develop over the course of three to four days [6]. Currently, there is no unanimous consensus on how to control or manage hemorrhagic complications associated with anticoagulation. Most guidelines, however, outline the immediate reversal of the anticoagulation through the use of vitamin K and factor replacement with either factor concentrates or fresh frozen plasma [6].

## Case presentation

A 64-year-old Caucasian female presented to the ED with complaints of coughing and shortness of breath for three days. Two days prior to admission, the patient contacted her primary care provider (PCP) via telehealth and stated that she had a persistent cough and had been unable to sleep. Her PCP gave appropriate medical recommendations to help provide her with symptomatic relief and told her to follow up if she did not feel better in three to four days. The patient denied any abdominal pain, nausea, vomiting, or other bleeding manifestations. The patient was eventually admitted for supratherapeutic international normalized ratio (INR, 10.06) and pneumonia and was kept NPO in anticipation of intervention. All other pertinent lab values on presentation to the ED are listed in Table 1.

**Table 1:** Pertinent abnormal lab values on presentation to the ED

	Presentation	Normal values
Hemoglobin (Hgb)	11.0	12.0-16.0 g/dL
Partial thromboplastin time (PTT)	68.1	25.0-35.0 s
International normalized ratio (INR)	10.06	2.0-3.0
Red blood cells (RBC)	3.83	4.22-5.4 x 10^4 cells/mcL
Serum glucose	322	70-110 mg/dL
HbA1c	8.2	=6.5%
Troponin	54	<40 ng/L

The patient has a past medical history of hypertension, type 2 diabetes, hyperlipidemia, prior pulmonary embolism, osteoarthritis, kidney stones, and gastroesophageal reflux disease. She endorses allergies to buspirone, citalopram, naproxen, and naproxen-esomeprazole. Her current medications include atenolol 25 mg QD, bismuth subsalicylate 262 mg/15mL q6 prn, bumetanide 0.5 mg QD, escitalopram 10 mg QD, hydrocodone-acetaminophen 5-325 BID, Januvia 100 mg QD, levothyroxine 100 mg QD, lisinopril 2.5 mg QD, metformin 500 mg four tablets QD, omeprazole 20 mg four tablets before each meal, oxycodone 15 mg q12, pioglitazone 30 mg QD, rosuvastatin 20 mg QD, and warfarin 4 mg one tablet Monday, Wednesday, Friday, Saturday, Sunday, and ½ tablet Tuesday.

Vitamin K was administered for stabilization of the supratherapeutic INR. When a repeat INR was done after treatment, it was within physiologic limits and had a value of 1.54. Additionally, she was administered ceftriaxone and azithromycin as treatments for her pneumonia infection. The patient’s rapid influenza A virus (IAV) test was positive, and the fecal occult blood test was negative. Chest X-ray showed “no acute pathology.” CT of the chest without contrast revealed a small left base infiltrate and left kidney stone without hydronephrosis.

**Figure 1:**
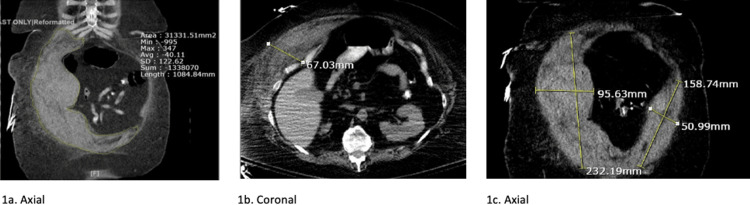
CT abdomen/pelvis with oral contrast only showing the following: “abnormal CT of the abdomen demonstrating large right anterior and lateral abdominal wall hematoma with acute and chronic components as described. No acute intra-abdominal or pelvic pathology is evident" (1a) Axial cut of CT illustrating the primary location of the hematoma in the right anterior and lateral abdominal wall. (1b) Coronal cut of CT confirming the location of the hematoma within the abdominal wall and illustrating the large size at an anteroposterior diameter of 67.03 mm. (1c) Axial cut of CT demonstrating the substantial size of the hematoma within the abdominal wall. Measuring its greatest size in the right lateral portion at 232.19 x 95.63 mm and in the left lateral portion at 158.74 x 50.99 mm.

On day two, the patient’s hemoglobin (Hgb) dropped from 11.0 to 6.6. This prompted a CT abdomen/pelvis with oral contrast and a blood transfusion, which brought the Hgb up to 7.4. The results of the CT abdomen/pelvis with only oral contrast, depicted in Figure 1, showed the following: “abnormal CT of the abdomen demonstrating large right anterior and lateral abdominal wall hematoma with acute and chronic components as described. No acute intra-abdominal or pelvic pathology is evident.”

The patient was promptly transferred to a tertiary care center for the expertise of interventional radiology (IR). However, after the transfer, the patient’s hematoma size stabilized, and she received further transfusions, resulting in her Hgb stabilizing. It was ultimately decided that intervention by IR to drain the hematoma was no longer necessary. After a short hospitalization, the patient was discharged to a rehabilitation facility, where she stayed for eight weeks before returning home.

## Discussion

Warfarin therapy in any patient has the ability to result in a hypercoagulable state. A rare but potentially life-threatening result of these supratherapeutic levels of warfarin is hematomas at various sites throughout the body. The most common are upper airway hematomas, sublingual, retropharyngeal, submaxillary, and epiglottal. However, even with the severe consequences of these events, there is no consensus in the medical community for diagnosis or management. This puts both patients and physicians in a challenging situation [7]. Most often, abdominal wall hematomas form from ruptures of the epigastric vessels. However, they may rarely be from deep circumflex iliac artery rupture or rupture of the rectus or lateral oblique muscles themselves [3].

Anticoagulation therapy, however, such as the use of warfarin, is not the only reason a patient may experience excessive bleeding. Often, viral infections are associated with the activation of general coagulation within the body. This increased coagulopathic state can thus lead to bleeding [8]. In a study looking at incidence and risk factors for rectus sheath hematomas, it was found that those most at risk were women of advanced age, in the context of an influenza or bronchitic process, who experienced fits of coughing [7]. In some infectious states, such as Ebola, activation of coagulation pathways may result in hemorrhagic fever. In IAV specifically, a higher incidence of cardiovascular events is noted, including both myocardial infarction and stroke. Moreover, although there are many causes of respiratory tract infections, IAV infection is common. Moreover, in many cases of influenza pneumonia in both patients and mice, alveolar hemorrhage has been reported [8].

Severe infection with the influenza virus represents a leading cause of global morbidity and mortality. Although the infection is thought to primarily cause pathology in the respiratory system, clinical reports also suggest that influenza infections are frequently associated with syndromes that involve many organ systems outside of the respiratory tract. A comprehensive literature review suggests that extrapulmonary complications, including cardio- and cerebrovascular events, myocarditis, CNS syndromes, and rhabdomyolysis, represent an underrecognized proportion of pathology in patients with influenza virus [9].

It is known that there is a high risk of hemorrhage associated with both anticoagulation, particularly warfarin, and influenza infection. However, there is also significant evidence to suggest a synergistic relationship between an increased risk of bleeding in patients with both warfarin anticoagulation and influenza infection. In fact, a study showed that mice given doses similar to the therapeutic doses used in patients infected with IAV and receiving a high dose of warfarin displayed an increased incidence of alveolar hemorrhage. Although low-dose warfarin did increase alveolar hemorrhage, it was not found to be statistically significant. Conversely, the use of warfarin did not increase alveolar hemorrhage in mice uninfected with IAV. The results of this study indicated that the administration of anticoagulants, such as warfarin, decreases the activation of coagulation and increases alveolar hemorrhage after IAV infection [8].

## Conclusions

Warfarin therapy is a long-used practice in the realm of anticoagulation. There are many clinical benefits to the use of this drug. However, as with any medication, there are also risks. Warfarin, in particular, has an increased risk for bleeding and eventual hematoma formation. Moreover, the incidence of hematoma formation in those infected with influenza is more common than one may initially think. The location of the hematoma varies greatly from patient to patient and can be very challenging to predict. However, knowing the vast amount of pressure created during a cough, especially in the abdominal muscles, it does not take a lot of imagination to understand how the formation of an abdominal wall hematoma would occur. Due to the great incidence of patients on warfarin therapy and the multitude of those infected with influenza and now COVID-19 each year, it is unimaginable how the incidence of hematomas in these patients is not more studied. This case brings to light the importance of close INR and clinical symptom monitoring and having a low threshold to suspect hemorrhage for those on warfarin therapy and with infections, such as influenza or COVID-19. A prompt diagnosis and the initiation of a standardized treatment plan must be made. Without this, escalation to a situation with life-threatening consequences for the patient is feasible.
